# Understanding and treating solid tumor–related disseminated intravascular coagulation in the “era” of targeted cancer therapies

**DOI:** 10.1177/2050312117749133

**Published:** 2017-12-21

**Authors:** Felice Vito Vitale, Giuseppe SA Longo-Sorbello, Stefano Rotondo, Francesco Ferrau

**Affiliations:** 1 Divisione di Oncologia Medica, Ospedale San Vincenzo, Messina, Italy; 2 Divisione di Ematologia, Ospedale San Vincenzo, Messina, Italy; 3 Ambulatorio Diagnostica Vascolare, Angiologia e Medicina Interna, IRCCS Piemonte-Bonino Pulejo, Messina, Italy

**Keywords:** Cancer, disseminated intravascular coagulation, solid tumors

## Abstract

A systemic activation of blood coagulation is usually present in many clinical conditions including the infectious or inflammatory ones and malignant disease as well. Depending upon circumstances, patients suffering from acute decompensated disseminated intravascular coagulation may be managed by a medical oncologist and either an internist or a physician working in an emergency and/or intensive care unit. In some cases, for example, the indolent ones, the activation of coagulation might not be easily detected by routine laboratory tests and not lead to clinical manifestations. Such a chronically activated intravascular coagulation can progress toward an overt decompensated disseminated intravascular coagulation. Traditional therapy of decompensated disseminated intravascular coagulation is based on reversing the underlying triggering disease and providing patients with adequate supportive treatment. The dilemma for the oncologist is whether or not the trigger cause can be treated and amended with a specific antineoplastic treatment, without worsening the consumption of platelets and the risk of bleeding. In light of the availability of new targeted therapies, the main criteria that should drive the strategy against solid cancer–related disseminated intravascular coagulation will be discussed.

## Introduction

Occurrence of decompensated disseminated intravascular coagulation is a major management challenge posed by cancer patients, particularly in the case of those suffering from solid tumors. The authors searched medical literature in their institutional libraries and PubMed. A number of peer reviewed articles deemed of relevant interest and published from 1983 to 2017 were taken into account for completion of this article. Medical oncologists are well aware that decompensated DIC is a potentially fatal complication often associated with the most aggressive types of tumor. Identification of patients who may be more likely to respond to a given anticancer drug should be the mainstay of treatment of cancer-related DIC. Whenever possible, a close cooperation between oncologist, hematologist and internist would be desirable.

## Discussion

Although the manuscript often highlights the personal authors’ point of view, the search of a link with evidence issued by several scientific papers published on the topic was constantly pursued. Therefore, this article may be considered a contribution to overcome a possible too pessimistic physicians’ attitude toward the treatment of solid tumor–related DIC.

### Understanding

Decompensated DIC may occur as the first sign of an underlying malignant disease or a late complication of a previously diagnosed and heavily treated cancer.^1,2^ Therefore, cancer patients suffering of such a disease may be initially admitted to hospitals under the care of physicians who belong to a division of Internal Medicine or of Clinical Oncology as well as an Emergency Care Unit and Intensive Care Unit. Physicians focusing their attention on these aspects may improve and hasten the diagnosis and start the best treatment. There is undoubted evidence that an interaction among coagulation/fibrinolysis pathways and cancer tissues exists.^3–5^ The interaction is mediated by an amount of molecules/enzymes such as cancer procoagulant (CP), tumor cell surface tissue factor (TF), microparticles carrying tissue factor, urokinase plasminogen activators (uPAs), plasminogen activator inhibitor-1 (PAI-1).^5–8^ Thus, cancer cells possess prothrombotic and fibrinolytic properties at once, and a thrombophilic state is present in almost all cancer patients.^9^ Accordingly, thromboembolic events and DIC, or coagulation consumption coagulopathy, can occur as result of the cancer-related prothrombotic tendency. Decompensated DIC is often present in patients who suffer from solid tumors or from hematological malignancy but with some different peculiarities. In fact, decompensated DIC frequently appears in early stage of some hematological malignancies while it mostly characterizes advanced or late stages of metastatic solid tumors.^2,10^ Among the solid tumor patients those harboring disseminated carcinomatosis of the bone marrow (DCBM) seem to be more susceptible to develop DIC.^11–13^ When decompensated DIC occurs in patients suffering from solid cancer, it is often associated with an indolent course: only a borderline or slowly dropping platelet count and a normal or slightly deranged level of other coagulation parameters such as prothrombin time (PT), activated partial thromboplastin time (APTT) and fibrinogen level.^14^ On the contrary, in most cases of hematologic malignancies, decompensated DIC presents itself as an acute consumption coagulopathy with rapid platelet count drop and coagulation factors exhaustion potentially leading to dramatic and fatal bleeding.^15^ However, bleeding is not the only life-threatening complication affecting DIC patients. Furthermore, widespread deposition of fibrin-rich thrombi in microvasculature and subsequent ischemia are both factors able to cause a fatal multiple organ dysfunction syndrome (MODS).^16–18^ The above type of thrombotic microangiopathy (TMA) observed in DIC course has a different pathogenesis in comparison with other TMAs as thrombotic thrombocytopenic purpura (TTP) or hemolytic uremic syndrome (HUS) which are usually not associated with coagulation factors consumption at least in their early stage despite presenting with thrombocytopenia.^17,19,20^ In clinical practice, many blood tests and a number of diagnostic guidelines are helpful to reveal and monitor the consumption coagulopathy associated with DIC along with its evolution and complications.^21–25^

### Treating

The approach to decompensated DIC varies from watchful waiting to active treatment according to the severity of clinical presentation. The treatment of the trigger causes at once with restoring (as necessary) coagulation factors and platelet count has a pivotal role in influencing the course of decompensated DIC.^21^ In any case, the main question that needs to be answered, especially in cancer patients, is whether or not the triggering cause can be amended; otherwise, every effort may be in vain. DIC is a clinicopathological syndrome characterized by a consumption coagulopathy potentially causing a decreased level of procoagulant proteins and platelets. For practical clinical application, DIC is classically divided into two stages: overt DIC (decompensated DIC) and non-overt DIC (not decompensated DIC).^22^ When decompensation occurs (overt DIC), it can manifest itself with different clinical and laboratory features depending on variable speed of platelets and coagulation factors depletion. In this regard, the efficiency of compensatory mechanisms (i.e. fibrinolysis activation or liver and bone marrow production of coagulation factors and platelets) along with the intensity of the underlying trigger conditions also affects the severity of the coagulopathy.^23^ A variety of pathological conditions, including solid tumors, play a causal role in initiating this coagulation disorder. Among solid tumors, especially adenocarcinomas are more prone to trigger both thromboembolic complications and consumption coagulopathy.^21^ Prevalence of overt DIC is estimated to be up to 7% in patients suffering from solid tumors.^2^ The course of an overt DIC can be unruly (acute DIC), dramatically translating to a serious thrombocytopenia and hypofibrinogenemia along with diffuse thrombosis within microvasculature or larger vessels, potentially leading to bleeding and MODS.^17^ However, in the case of solid tumors, the coagulopathy has, most often, an indolent presentation and chronic course that may also be defined as low intensity decompensated DIC or mild DIC. It is less commonly associated with hemorrhagic complications and characterized by minimally deranged blood coagulation parameters. Therefore, DIC can produce laboratory signs alone or in conjunction with clinical symptoms.^15^ In general, treatment of the underlying disease is the instrumental key in reversing the consumption coagulopathy and, in addition, supportive therapy based on blood component infusion and/or low dose heparin and/or antifibrinolytics could be started simultaneously.^21^ The administration of heparin should be considered in non-symptomatic DIC, namely without bleeding, and low molecular weight heparin should be preferred over unfractionated heparin.^24,25^ Unfortunately, the removal of the trigger cause of DIC was and still is often impossible when physicians deal with metastatic and/or heavily pretreated solid tumors. In fact, cancer is not a self-limiting disease and is not always successfully manageable. Current available guidelines provide recommendations for optimal use of blood components such as fresh frozen plasma (FFP) and platelet concentrate, and, where appropriate, heparin and/or antifibrinolytics-based therapy in DIC irrespectively of its causal factors.^24,25^ FFP infusion aims to correct alterations of coagulation parameters resulting from plasma coagulation factors depletion; heparin is commonly used in order to inhibit or slow down the coagulation cascade; antifibrinolitics might have a role in treating DIC with hyperfibrinolysis and bleeding dominant complication.^21,25^ Administration of blood components is mandatory in the event of active bleeding and platelet count of <50 × 10^9^/L, or if the perceived risk of bleeding is increased (e.g. in patients requiring invasive procedures who present prolonged PT/APTT (greater than 1.5 times normal), decreased fibrinogen and a platelet count of <20 × 10^9^/L or sometimes higher).^24,25^ It is interesting to note that supportive therapy other than and in addition to traditional treatments can be considered for patients with both DIC and DCBM. Bisphosphonates (i.e. zoledronic acid) or denosumab based supportive treatment is considered able to inhibit or slow down tumor-related bone resorption and can benefit patients with DCBM.^13^ Basically, in the context of solid tumors, the main controversy is not so much the appropriate indication for using blood component therapy but whether the treatment of the tumor triggering the coagulopathy is worth being undertaken or not. In fact, the blood component administration alone or in conjunction with heparin or, if it is the case, with antifibrinolytics is usually not able to stop and/or reverse DIC in cancer patients in the absence of an effective treatment of the underlying malignant disease.^26^ Two key issues should be addressed when the oncologists/physicians are dealing with patients affected from cancer-related DIC:

1. Should we only treat the clinical and laboratory manifestations of the coagulopathy or the tumor (that is the trigger cause) as well? In other words, when is a given tumor causing DIC worth being treated?
2. In addition, should the malignant disease not be suitable for an effective treatment, what would the aim of administering blood component therapy be?

In essence, the decision whether to treat the cancer or not is mainly influenced by two factors: one lies in the cancer potential responsiveness to a specific therapy and another in the expected therapy-related myelotoxicity. Additional key factors are listed in Table 1. An acute DIC course is a clinical condition rapidly worsening and requiring urgent and specific supportive treatments. It is characterized by severe thrombocytopenia in conjunction with coagulation factors exhaustion frequently leading to ongoing emorrhage and anemia.^25^ Conversely, when DIC has an indolent/chronic course, the clinical picture is essentially oligo or asymptomatic and no urgent treatments may be required over a long period of time.^14,21,25^ As to anticancer treatments, a large amount of data regarding the toxicity and response rate of any anticancer drug are usually published in several scientific papers and can be easily used in clinical practice. The term terminally ill cancer patients essentially identify a population whose clinical conditions progressively worsen with a life expectancy of 6 months or less and no treatments are able to restore their health.^27^ Regarding the approach to the clinical and/or laboratory manifestations of DIC, the choice of starting supportive measures (blood component therapy, heparin, etc) is mainly based on the course (indolent/chronic or acute) of the consumption coagulopathy. If no effective therapy should be available to ensure an effective treatment of underlying malignancy, this is for example the case of terminally ill cancer patients, the above-mentioned supportive therapies would have an essential role in preventing patients from uncomfortable physical and psychological clinical complications such as external bleeding.^28^ Of course, the current general guidelines can help in deciding which supportive measures are more appropriate for the patients.^24,25^ Decompensated DIC usually, but not always, occurs in the advanced or later stages of solid cancer.^2,29^ At the opposite, it is well known that most hematological malignancies are remarkably chemosensitive and, at least in the early cancer stage, the related DIC is more successfully treated than when it occurs in the solid ones.^30^ Accordingly, the DIC occurring during hematological diseases can regress more easily than expected in the course of solid tumors. Thus, only a small percentage of patients suffering from solid cancer and decompensated DIC are suitable candidates for and take advantage of chemotherapy. This is the case of some histotypes, that is, gastric cancer or breast cancer, considered very chemoresponsive.^2,31,32^ As a result, over the past decades, the oncologists who treat solid tumors developed an almost defeatist approach to cancer-related DIC. Sallah et al. had already pointed out the relevance of “patient’s performance status and prior therapy” to make the right decision whether or not to treat the underlying malignancy.^2^ To date, in addition, other factors should be taken into account before deciding to start a therapy specifically directed to the cancer in patients with DIC. For example, tumor’s histotype in conjunction with additional biological characteristics has to be considered among the main factors influencing the “expected tumor response to anticancer therapy.” Fortunately, at present, many genotype-driven and/or targeted therapies, often less myelotoxic and more effective than chemotherapy, are taking place in the therapeutic armamentarium of the oncologists.^33–35^ In this regard, the effectiveness of some of the new anticancer drugs is very impressive. The oncologists have now the option of targeted therapies with fast response as B-raf/MEK inhibitors in malignant cutaneous melanoma or epidermal growth factor receptor (EGFR) inhibitors in EGFR exon 19 and 21 gene mutation or anaplastic lymphoma kinase (ALK) inhibitors in EML4-ALK gene re-arrangement in patients affected from non-small cell lung cancer.^33,36^ These new anticancer drugs may reconcile a low myelotoxicity with high specificity and response rate even against aggressive tumors often involved in triggering DIC.^37^ The above characteristics might make them attractive for the oncologists when approaching frail cancer patients even if affected from tumor-related decompensated DIC and subsequent thrombocytopenia. Some authors recently reported a really remarkable regression of acute DIC and cancer dissemination in poor performance status patients with non-small cell lung cancer who were administered erlotinib (an EGFR inhibitor) and crizotinib (an ALK inhibitor).^38,39^ Overall, Table 1 summarizes factors that influence treatment decision making in solid tumor–related DIC and itemize arguments for and against therapeutic interventions tailored to specific tumor characteristics (e.g. histological, biological, genetic) in conjunction with supportive measures (e.g. platelet and FFP or red blood cell transfusion plus or less heparin or antifibrinolytics).

**Table 1.** Arguments PROS or CONS treatment of decompensated DIC in solid tumors inclusive of a specific anticancer therapy.

	PROS	CONS
DIC course	Acute	Indolent
Expected response rate to anticancer therapy	High	Low
Incidence of anticancer therapy induced myelotoxicity	Low	High
Terminally ill cancer patient	Not	Yes

DIC: disseminated intravascular coagulation.

## Conclusion

The single patients’ and tumors’ characteristics, along with DIC course, are the main criteria to dictate a therapeutic choice possibly including specific anticancer drugs. The current availability of a number of targeted therapies may open out new opportunities of effectively treating the cause of the cancer-related consumption coagulopathy. As a result, the outcome of DIC might be improved even in rapidly progressive tumors. Accordingly, a comprehensive approach to a consumption coagulopathy should include an as much as possible accurate biological characterization of the tumor and the choice of the most specific and less myelotoxic anticancer treatment if any. Of course, a close multidisciplinary cooperation among oncologist, hematologist, and internist should be required in most cases and the best treatment option discussed on an individual basis.
